# What do Australian adults eat for breakfast? A latent variable mixture modelling approach for understanding combinations of foods at eating occasions

**DOI:** 10.1186/s12966-021-01115-w

**Published:** 2021-03-25

**Authors:** Rebecca M. Leech, Carol J. Boushey, Sarah A. McNaughton

**Affiliations:** 1 Institute for Physical Activity and Nutrition (IPAN), School of Exercise and Nutrition Sciences, Deakin University, Geelong, Victoria Australia; 2 Epidemiology Program, University of Hawaii Cancer Center, Honolulu, HI USA

**Keywords:** Eating patterns, Dietary patterns, Eating occasions, Breakfast, 24-h recall

## Abstract

**Background:**

The patterning of food intake at eating occasions is a poorly understood, albeit important, step towards achieving a healthy dietary pattern. However, to capture the many permutations of food combinations at eating occasions, novel analytic approaches are required. We applied a latent variable mixture modelling (LVMM) approach to understand how foods are consumed in relation to each other at breakfast.

**Methods:**

Dietary intake at breakfast (*n* = 8145 occasions) was assessed via 24-h recall during the 2011–12 Australian National Nutrition and Physical Activity Survey (*n* = 3545 men and *n* = 4127 women, ⩾19 y). LVMM was used to determine breakfast food profiles based on 35 food group variables, reflecting compliance with Australian Dietary Guidelines. F and adjusted-chi2 tests assessed differences in timing of consumption and participant characteristics between the breakfast profiles. Regression models, adjusted for covariates, were used to examine associations between breakfast food profiles and objective adiposity measures (BMI and waist circumference).

**Results:**

Five distinct profiles were found. Three were similar for men and women. These were labelled: “Wholegrain cereals and milks” (men: 16%, women: 17%), “Protein-foods” (men and women: 11%) and “Mixed cereals and milks” (men: 33%, women: 37%). Two “Breads and spreads” profiles were also found that were differentiated by their accompanying beverages (men) or type of grain (women). Profiles were found to vary by timing of consumption, participant characteristics and adiposity indicators. For example, the “Protein-foods” profile occurred more frequently on weekends and after 9 am. Men with a “Bread and spreads (plus tea/coffee)” profile were older (*P* < 0.001) and had lower income and education levels (*P* < 0.05), when compared to the other profiles. Women with a “Protein-foods” profile were younger (P < 0.001) and less likely to be married (*P* < 0.01). Both men and women with a “Wholegrain cereals and milks” profile had the most favourable adiposity estimates (*P* < 0.05).

**Conclusions:**

We identified five breakfast food profiles in adults that varied by timing of consumption, participant characteristics and adiposity indicators. LVMM was a useful approach for capturing the complexity of food combinations at breakfast. Future research could collect contextual information about eating occasions to understand the complex factors that influence food choices.

**Supplementary Information:**

The online version contains supplementary material available at 10.1186/s12966-021-01115-w.

## Introduction

A poor quality diet is a major modifiable contributor to obesity and its long-term effects, including chronic disease death and disability, globally [1]. Current dietary guidelines around the world utilise a food-based framework to promote the adoption of healthy dietary patterns [2–4]. The emphasis by dietary guidelines on foods that make up the whole diet, rather than on individual nutrients, reflects the increasing body of evidence that has accrued over recent decades in relation to the health effects of specific food groups and dietary patterns [5, 6]. Nutrition guidance based on foods is also more relevant because people select foods, rather than nutrients, and overall diet quality is thus driven by the quality of food components.

However, individuals rarely consume foods in isolation. Instead, they select combinations of foods at eating occasions, including meals and snacks. The patterning of food intake at eating occasions, or eating patterns, are an integral, yet poorly understood, intermediary step to achieving a healthy dietary pattern and preventing obesity and its complications [7]. Eating patterns incorporate aspects of timing, frequency, regularity, as well as, the food and nutrient composition of eating occasions [8]. There is growing recognition and research to suggest that aspects of eating patterns play an important role in weight management and health [7, 9]. Furthermore, an understanding of eating patterns can inform the development and translation of dietary guidelines by helping to contextualise advice and provide practical guidance on what foods should be consumed more often, in which combinations and at which times [10, 11].

A key aspect of eating patterns is the composition of eating occasions, particularly the combination of foods [7]. The breakfast eating occasion has long been marketed as “the most important meal of the day” [12] and evidence suggests that regularly eating breakfast promotes better overall diet quality [7, 9] and, possibly, more favourable cardiometabolic risk factors including obesity [9, 13]. For example, there is some evidence that regular consumption of ready-to-eat breakfast cereals may protect against weight gain, although the effects of different breakfasts, including cereal types remains unclear [14, 15]. Research that provides a detailed examination of food combinations at eating occasions in relation to obesity and health outcomes is needed to elucidate meal-specific diet–disease associations. Further, dietary guidance for what to consume at breakfast and other eating occasions are usually provided as a list of food groups or components to include or limit but does not specify the possible ways foods may be selected and combined to optimise diet quality. Eating patterns may be influenced by national and cultural norms [16]; and dietary guidance that integrates nationally and culturally relevant food practices may also assist populations to follow a healthy eating pattern.

Relatively little research has examined how people select and combine foods at eating occasions, [7], including at breakfast [17–19]. One reason for this is the analysis of eating occasions is challenging due to the skewed and semi-continuous nature of the food intake data. This is explained by recognizing that an eating occasion is a snapshot of time during which a relatively small number of foods are selected for consumption from a larger array of food choices resulting in many possible food combinations and the possibility of a high proportion of non-consumption for a given food subgroup at each eating occasion. Few approaches to overcome these data challenges have been explored; these include data mining techniques [17, 18] and semiparametric Gaussian Copula Graphical models [19]. While these exploratory approaches aid understanding of complex interrelationships between dietary variables from multi-dimensional data, they do not characterize individuals on the basis of their dietary behaviours by either designation of a dietary score or group membership (i.e., dietary patterns analysis) [20]. Further exploration of alternative approaches that are accessible and feasible for understanding how individuals consume foods in relation to each other at eating occasions will help disentangle the patterns of consumption at eating occasions and opportunities for achieving a healthy eating pattern.

Cluster analysis, and more recently latent class analysis, are two established exploratory statistical approaches that can be useful for understanding the patterning of behaviours [21–23], including dietary patterns [6, 20, 24, 25] and temporal eating patterns [26, 27]. Since the approaches are data-driven and person-centred, they can be used to identify different groups of people who share similar patterns of behaviour. An advantage of LCA over cluster analysis is the derived groups (referred to as clusters, classes or profiles) are based on probabilistic mixture modelling which helps to minimise the subjectivity regarding the selection of final group numbers and can be extended to handle complex data (e.g., binary, categorical, ordinal, continuous variables with standard and non-standard distributions and skewness) [28]. Therefore, we applied a latent variable mixture modelling (LVMM) approach (an extension of LCA) to examine profiles of food combinations at breakfast eating occasions in a large and nationally representative sample of Australian adults. To understand the potential use of LVMM for assessing meal-specific associations with obesity and health outcomes, we also examined the whether the resulting breakfast profiles varied by time of consumption and participant characteristics, including adiposity.

## Subjects and methods

### Sample and study design

This study is a secondary cross-sectional analysis of nationally representative dietary data collected from adult respondents who participated in the Australian National Nutrition and Physical Activity Survey, 2011–2012 (NNPAS 2011–12). The NNPAS, administered by the Australian Bureau of Statistics (ABS), covered approximately 97% of the population living in urban and rural (excluding very remote) areas of Australia; the survey design and data collection methods have been published in detail previously [29]. In summary, the survey employed a multistage, probability sampling design of usual residents in private dwellings, and included 12,153 persons aged 2 years or over (77% response rate) of whom 9115 were adults (*n* = 4282 men and *n* = 4833 women) aged over 18 years, not currently pregnant or breastfeeding. Person-specific weights, adjusted for probability of selection and non-response, were used to provide estimates relating to the whole population. The Census and Statistics Act 1905 provides ethics approval for the ABS to conduct the household interview components of health surveys [29]. As this study involves the secondary analysis of pre-existing and non-identifiable data, an exemption from ethics review was approved by the Deakin University Human Research Ethics Committee ([DUHREC]; application 2018–415).

### Dietary assessment

Dietary data were collected during two 24-h recalls using the validated USDA automated multiple 5-pass method [30, 31]. The dietary assessment period covered all four seasons and all days of the week (excluding Sunday, unless requested by the participant) between May 2011 and June 2012. The distribution of recalls across the week were as follows: between 14 and 18% for weekdays, 11.5% for Saturdays and 3.5% for Sundays [29]. The first recall was conducted by trained ABS staff during a household interview and the second was conducted approximately 9 days later during a telephone interview in a subset of 6053 (65%) of the adult respondents. For the current exploratory analysis of breakfast eating occasions, nationally-representative dietary data from men and women who completed the first recall day were used.

### Breakfast eating occasions and food groups

During the 24-h recall, respondents identified the starting time and type of each eating occasion. A single eating occasion was defined according to the current recommendations as any occasion where a food or beverage was ingested, contained a minimum energy content of 210 kJ and was separated in time from the preceding and succeeding eating occasions by 15 min [32]. Eating occasions reported by the participant as breakfast/brunch were coded as breakfast, consistent with recent research examining the nutritional composition of breakfast in US adults [33–35]. As shown in Additional File 1, participants were excluded from the analysis if they were missing data on time of eating (*n* = 226), did not report a breakfast eating occasion (*n* = 956) or reported a breakfast eating occasion containing fewer than 210 kJ (*n* = 196).

All foods and beverages reported by participants at breakfast (*n* = 8145 eating occasions; Additional File 1) were classified into 35 food groups (described in Additional File 2) to align with the 2013 Australian Dietary Guidelines (ADG) [2]. Food and beverage intakes (g/d) were calculated using the Australian Health Survey – ADG database [36] and the AUSNUT 2011–13 food group classification [37], both developed by Food Standards Australia New Zealand for the NNPAS 2011–12. For the ADG Database, the ABS disaggregated and estimated all individual food components within a mixed-dish using the AUSNUT 2011–13 recipe file [37].

Using the ADG database [36], intakes (g/d) of 26 food groups consumed by participants at breakfast were calculated. These included 22 groups based on the five food group foods: grains (6 groups), vegetables and legumes (5 groups), fruit (excluding fruit juice; 2 groups), dairy and alternatives (4 groups) and meat and alternatives (5 groups). Other ADG groups included unsaturated spreads and oils (2 groups), water (1 group) and 100% fruit juice (1 group). Intake of food groups (g/d) not estimated in the ADG database were calculated based on the AUSNUT 2011–13 (minor and major food codes) food group classification system [37]. These included tea/coffee (one group) and energy-dense nutrient poor ‘discretionary’ foods (8 groups) [38]. All food group intakes at the breakfast eating occasion were adjusted for breakfast energy intake using the residual method by regressing food group intakes on total energy intake at breakfast [39, 40].

### Time of consumption

Using information on the day of the week that the 24-h dietary recall was administered, breakfast occasions were classified as occurring between Monday and Friday (weekday) or on Saturday/Sunday (weekend). Breakfast occasions were further classified according to the following time slots: < 7:00 h, 7:00–7:59 h, 8:00–8:59 h and ≥ 9:00 h, using information on eating occasion starting time.

### Participant characteristics

During the household interview, information was also collected about participants’ age, gender, country of birth, geographical region of residence, social marital status, highest attained level of education, and household income [29]. Unless indicated otherwise, the variables used for this analysis were defined and categorised by the ABS and included: country of birth (Australia, other ‘mainly English speaking’ countries, all other countries); geographical region of residence (Major cities of Australia, inner regional cities of Australia, other regions); social marital status (married [in a registered or de facto marriage], not married); smoking status (never smoked, past-smoker, current smoker), and meeting (yes or no) the Australian physical activity guidelines of 150 min and 5 sessions in the previous week [41]. Highest attained level of education was provided by the ABS and further categorised for this analysis as low (completed some high-school or less), medium (completed high-school or completed some high-school and/or certificate/diploma) or high (having a tertiary qualification). Deciles of participants’ weekly gross household income (Australian dollars [AUD]) that accounted for the number of persons living in the household were provided by the ABS [29]. For this analysis, deciles were collapsed into quintiles (reference ranges in AUD: quintile 1, <$398; quintile 2, $399–638; quintile 3, $639–958; quintile 4, $959–1438 and quintile 5, ≥ $1439). Participants reported the number of hours worked in the previous week and were categorised as: 0 h, < 40 h or ≥ 40 h per week. Anthropometric measurements (to 1 decimal point) of height (cm; portable stadiometer), weight (kg; digital scales), and waist circumference (cm; metal tape measure) were taken by trained ABS staff during the household interview. Body mass index (BMI) (kg/m2) was calculated.

### Statistical analysis

#### Latent profiles of breakfast food combinations

Two-part latent Variable Mixture Modelling (LVMM) was performed in M*Plus* Version 8.2 (Muthen & Muthen, Los Angeles, CA, USA) to identify distinct breakfast food combination profiles for men and women, separately. LVMM is an extension of latent class analysis, a statistical technique that identifies categorical latent class/profile variables on the basis of observed categorical or continuous variables [21, 42]. For this analysis, the data “two-part” option was applied in M*Plus* as the intake distributions of the 35 food groups at breakfast were semi-continuous (i.e., had a lumping of values at zero and a highly-skewed tail) [43]. The two-part option allows for estimation of both the binary distribution of the variable (i.e. was the food group consumed or not consumed at breakfast) and the continuous distribution of the variable (i.e. mean intake among the food group consumers). Therefore, participants were assigned to the latent profiles on the basis of: 1) consumption of food groups at breakfast (yes/no) and 2) the intake amount of food groups consumed (if consumed). Although a small number of participants (*n* = 230) reported more than one breakfast eating occasion, these participants were included for the identification of food combinations and the “complex mixture” analysis option was specified to allow for the clustering of eating occasions by individuals [42, 43]. The NNPAS replicate weights were not applied as this option was not available with the complex mixture analysis in M*Plus*. The final number of latent profiles to extract was determined using model fit indices. These included: the Bayesian information criterion (BIC) and sample size adjusted BIC (lower values indicate a better model fit); the standardised entropy index (value range between zero and one; higher values indicate better class separation), and the Lo-Mendell-Rubin Likelihood Ratio test [LMR-LRT], which compares nested (k vs. k-1) latent class models using an approximation of the distribution of difference of the two log likelihoods. A *p*-value < 0.05 for the LMR-LRT indicates that the more complex model (k) provides a better fit for the data than the simpler model (k-1). The interpretability of the breakfast food profiles was also considered when determining the final latent profile solution (Table 1) [42, 43]. As there may be day-to-day variability in breakfast food profiles (i.e., a breakfast food profile may differ from 1 day to the next), the reliability of the latent profiles was examined by repeating LVMM procedures using data from the second day of dietary recall (*n* = 2320 men and *n* = 2770 women).

**Table 1:** Model fit indices for latent profiles of food combinations at breakfast eating occasions^a^

	2 classes	3 classes	4 classes	5 classes	6 classes
*Men*					
Loglikelihood	−60,711.023	−59,309.719	−58,281.212	−57,825.658	− 57,424.792
Entropy	0.819	0.873	0.872	0.868	0.849
BIC	122,871.330	120,642.489	119,181.015	118,854.561	118,637.483
adjusted BIC	122,312.086	119,857.641	118,170.563	117,618.505	117,175.822
^b^LMR-LRT	5586.967, *P* < 0.001	2808.885, *P* < 0.001	2042.776, *P* < 0.001	912.682, *P* = 0.01	794.951, *P* = 0.08
*Women*					
Loglikelihood	−67,230.626	−65,773.386	−64,449.367	−63,598.615	−63,454.789
Entropy	0.863	0.881	0.878	0.926	0.889
BIC	135,936.216	133,617.594	131,564.811	130,458.564	130,766.168
adjusted BIC	135,376.959	132,832.727	130,554.335	129,222.479	129,304.474
^b^LMR-LRT	6122.822, *P* = 0.12	2900.629, *P <* 0.0001	2643.024, *P* < 0.0001	1742.887, *P* < 0.0001	336.794, *P* = 0.13

^a^*AIC* Akaike Information Criterion, *BIC* Bayesian Information Criterion, *BS* Bootstrap, *LMR* Lo-Mendell-Rubin, *LRT* likelihood ratio test

^b^Adjusted Lo-Mendell-Rubin likelihood ratio test for k versus k-1 profiles. Values are two times the loglikelihood difference and corresponding *p*-value

#### Associations between latent breakfast profiles, time of consumption and participant characteristics

All analyses for the associations of the breakfast food profiles with participant characteristics were conducted using Stata 15 (Stata Inc., College Station, TX, USA). The analysis used person weights to derive point estimates relating to the Australian population and replicate weights to adjust standard errors for the clustered survey design [29]. For all analyses, *P* < 0.05 was considered statistically significant. The very small proportion (2.8%) of participants reporting more than one breakfast eating occasion were excluded from the subsequent analyses of associations with food intakes and participant characteristics which included 3545 men and 4127 women.

Descriptive statistics for food intakes, time of consumption and participant characteristics are presented as weighted means (95% confidence intervals [CI]) or weighted percentages. As some foods were rarely consumed for a given breakfast food profile, the mean intake was only calculated when the proportion of participants consuming the item was ≥5%. For continuous variables, the *F-*test was used to determine differences in participant characteristics between latent breakfast food profiles, with Bonferroni correction to account for multiple testing across > 2 profiles. For categorical variables, differences between latent profiles were assessed using the adjusted Pearson Chi-2 test for survey data. Multiple linear regression (for continuous outcomes) and logistic regression (for binary outcomes) were used to test for associations between breakfast food profiles, and the following measures of adiposity: BMI and waist circumference (WC; continuous) and overweight/obesity and central overweight/obesity status (binary). Models included men and women with no missing data for BMI and WC. All adiposity estimates were adjusted for age (y, continuous), education level (categorical), smoking status (categorical), meeting physical activity guidelines (categorical) and energy intake (kJ, continuous) at all eating occasions excluding breakfast. Pairwise differences, with Bonferroni correction, of adjusted mean adiposity estimates between latent breakfast profiles were calculated.

## Results

### Latent profiles of breakfast food combinations

Model fit indices favoured a five-class model for both men and women (Table 1). The combination of smaller BIC and adjusted BIC values, entropy values > 0.8 for and results from the LMR-LRT indicated a five-class solution. The breakfast food profiles for men and women were labelled according to their distinguishing features, indicated by the proportion of participants consuming particular food group items and the corresponding mean intake at the breakfast eating occasion, when compared to the other profiles (Tables 2 and 3). Similar breakfast food profiles were also observed after repeating LVMM procedures on the second day of dietary recall data (Additional Files 3 and 4). Three profiles were similar for men and women. These included a: “Wholegrain cereals and milks” profile (men: 16%, women: 17%) as almost all participants with this profile reported consuming wholegrain high fibre cereals. Other foods that featured in this profile, compared to other profiles, were low fat milks, dried fruit, fresh/canned fruit, nuts and seeds and yoghurts/custards. The profile, labelled “Protein-foods”, (men; 11% and women: 11%) was characterised by intakes of eggs, processed meats and refined grains or bread and unsaturated oils. Cheeses featured more strongly in the profile and this was the only profile where vegetables, including legumes were represented. The third similar profile for men and women, labelled “Mixed cereals and milks” (men: 33%, women: 37%) featured a mix of cereals types (e.g., wholegrain cereals, refined grain cereals and sweetened cereal products) and milks. Fresh fruit was also reported by 19 and 30% of men and women with this profile, respectively. Two further profiles, labelled “Breads and spreads 1” (men: 23%, women: 18%) and “Bread and spreads 2” (men: 15%, women: 17%) were characterized by breads (wholegrain and/or refined grain) and spreads (discretionary and/or unsaturated). However, these two profiles were differentiated by their accompanying beverages (men) or type of grain (women). For example, 90% of men with a “Breads and spreads 1” profile reported consuming coffee or tea whereas sugar-sweetened beverage intake consumption was highest for the “Breads and spreads 2” profile. Among women with a “Bread and spreads 1” profile, almost all women consumed wholegrain breads whereas refined grain breads were consumed by all women with the “Breads and spreads 2” profile. Added sugar featured most strongly in profiles with the highest tea or coffee consumption (i.e., “Breads and Spreads 1” for men and “Breads and spreads 2” for women). Complete data on the intake of all 35 food group variables, overall and by latent breakfast profile, are provided in Additional Files 5 and 6.

**Table 2:** Energy-adjusted intakes of the key food groups at the breakfast eating occasion according to latent breakfast profile among Australian men (*n* = 3545)^a^

	Percentage (%) of consumers	Mean (95% CI) intake (g) among consumers
***Profile 1: “Wholegrain cereals and milks”***	***16***	
Food group (g)		
Wholegrain high fibre cereals	99	38.4 (36.3, 40.6)
Dried fruit	87	10.0 (9.2, 11.0)
Reduced fat milks	51	127.3 (112.8, 143.8)
Tea or coffee	51	206.9 (186.9, 229.2)
Nuts and seeds	45	5.3 (4.7, 5.9)
Medium fat milks	38	106.1 (93.4, 120.5)
Fresh or canned fruit	37	41.9 (31.9, 55.1)
Unsaturated oils	30	2.1 (2.0, 2.2)
Water	24	574.2 (460.8, 715.4)
Yoghurts and custards	20	64.8 (57.1, 73.5)
***Profile 2: “Protein-foods”***	***11***	
Food group (g)		
Unsaturated oils	69	3.9 (3.5, 4.3)
Eggs	61	54.9 (48.2, 62.6)
All other vegetables	51	34.5 (28.5, 41.7)
Tea or coffee	50	235.9 (217.4, 256.0)
Refined grain low fibre breads	49	49.7 (43.8, 56.5)
Processed meats	43	35.7 (32.0, 39.8)
Unsaturated spreads	43	1.9 (1.6, 2.1)
Brassica vegetables	33	13.6 (10.9, 16.9)
Medium fat milks	28	39.0 (32.5, 46.7)
Cheeses	25	16.6 (13.7, 20.1)
Water	21	687.1 (503.2, 938.2)
Condiments	20	16.4 (13.7, 19.5)
Refined low fibre grains	15	17.5 (8.0, 38.6)
***Profile 3: “Breads and spreads 1”***	*** 23***	
Food group (g)		
Tea or coffee	90	205.6 (192.9, 219.1)
Discretionary spreads	65	12.2 (11.1, 13.4)
Refined grain low fibre breads	51	59.1 (54.8, 63.8)
Sugar	50	7.7 (7.2, 8.4)
Whole grain high fibre breads	46	55.7 (52.3, 59.2)
Medium fat milks	46	52.1 (46.3, 58.5)
Unsaturated spreads	44	9.0 (8.3, 9.7)
Reduced fat milks	28	53.6 (46.4, 62.0)
Water	16	637.5 (483.3, 844.2)
***Profile 4: “Mixed cereals and milks”***	*** 33***	
Food group (g)		
Medium fat milks	50	154.6 (143.6, 166.5)
Tea or coffee	44	236.6 (221.9, 252.3)
Whole grain high fibre cereals	38	48.6 (46.2, 51.2)
Sugar	37	10.8 (10.0, 11.6)
Reduced fat milks	32	159.2 (140.3, 180.7)
Fresh or canned fruit	19	100.3 (80.2, 125.4)
Water	19	692.4 (564.2, 849.6)
Whole grain high fibre grains	17	191.0 (169.5, 215.3)
Refined grain low fibre cereals	17	36.0 (32.9, 39.4)
***Profile 5: “Breads and spreads 2”***	*** 15***	
Food group (g)		
Refined grain low fibre breads	41	65.5 (61.0, 70.4)
Discretionary spreads	39	10.6 (8.9, 12.7)
Wholegrain high fibre breads	34	67.6 (62.0, 73.8)
Unsaturated spreads	25	8.8 (7.2, 10.7)
Water	24	1080.2 (792.0, 1473.2)
Unsaturated oils	17	2.8 (2.3, 3.3)
Sugar-sweetened beverages	16	167.7 (110.1, 255.5)

^a^Values shown are weighted percentage (%) of women who reported consuming one or more food/beverage items from each food group and weighted geometric mean (95% confidence interval) intakes (g) of energy-adjusted food groups

**Table 3:** Energy-adjusted intakes of the key food groups at the breakfast eating occasion according to latent breakfast profile among Australian women (*n* = 4127)^a^

	Percentage (%) of consumers	Mean (95% CI) intake (g) among consumers
***Profile 1: “Wholegrain cereals and milks”***	***17***	
Food group (g)		
Wholegrain high fibre cereals	99	37.3 (35.7, 38.9)
Dried fruit	86	9.8 (9.1, 10.5)
Reduced fat milks	59	109.3 (99.4, 120.2)
Tea or coffee	55	223.6 (202.1, 247.5)
Nuts and seeds	49	5.4 (4.9, 6.0)
Fresh or canned fruit	41	37.7 (29.6, 48.0)
Medium fat milks	25	99 (84.1, 116.7)
Yoghurts and custards	25	65.0 (57.5, 73.5)
Water	23	642.1 (504.0, 818.2)
***Profile 2: “Protein-foods”***	***11***	
Food group (g)		
Unsaturated oils	56	3.9 (3.4, 4.4)
All other vegetables	46	31.4 (25.0, 39.4)
Eggs	43	56.0 (47.9, 65.5)
Tea or coffee	41	220.6 (192.9, 252.3)
Refined grain low fibre breads	36	51.3 (45.2, 58.2)
Unsaturated spreads	36	2.1 (1.7, 2.6)
Medium fat milks	29	43.2 (31.5, 59.3)
Brassica vegetables	28	16.0 (11.4, 22.4)
Refined low fibre grains	26	15.7 (10.3, 23.9)
Processed meats	26	29.1 (25.8, 32.8)
Water	24	850.2 (670.41078.3)
Orange vegetables	22	11.5 (8.0, 16.4)
Starchy vegetables	21	13.5 (7.2, 25.2)
Cheeses	18	18.5 (15.5, 22.0)
***Profile 3: “Breads and spreads 1”***	***18***	
Food group (g)		
Whole grain high fibre breads	99	55.5 (53.5, 57.6)
Tea or coffee	66	211.3 (199.7, 223.6)
Discretionary spreads	60	10.7 (9.7, 11.8)
Unsaturated spreads	39	6.6 (6.0, 7.3)
Reduced fat milks	35	55.3 (48.1, 63.7)
Medium fat milks	22	60.0 (48.7, 73.9)
Sugar	22	5.8 (5.2, 6.4)
Water	16	745.7 (584.9, 950.7)
***Profile 4: “Mixed cereals and milks”***	***37***	
Food group (g)		
Tea or coffee	51	221.2 (208.2, 235.1)
Reduced fat milks	38	130.6 (119.2, 143.1)
Medium fat milks	37	140.6 (128.4, 153.9)
Fresh or canned fruit	30	87.6 (75.7, 101.4)
Whole grain high fibre grains	27	192.0 (180.3, 204.4)
Sugar	27	8.9 (8.1, 9.8)
Whole grain high fibre cereals	24	42.2 (40.4, 44.1)
Water	23	657.0 (556.8, 775.3)
Refined grain low fibre cereals	14	36.0 (33.8, 38.3)
***Profile 5: “Breads and spreads 2”***	***17***	
Food group (g)		
Refined grain low fibre breads	100	55.2 (52.8, 57.8)
Tea or coffee	68	215.3 (193.4, 239.6)
Discretionary spreads	62	11.8 (10.7, 12.9)
Unsaturated spreads	36	7.9 (7.2, 8.7)
Sugar	30	6.9 (6.3, 7.5)
Reduced fat milks	27	46.5 (39.1, 55.3)
Medium fat milks	24	60.4 (52.0, 70.2)
Water	17	567.2 (430.8, 746.7)

^a^Values shown are weighted percentage (%) of women who reported consuming one or more food/beverage items from each food group and weighted geometric mean (95% confidence interval) intakes (g) of energy-adjusted food groups

### Time of consumption of latent breakfast food profiles

The consumption of breakfast food profiles for men and women differed by day of the week (weekend v weekday) and time-of-day (Table 4). In both men and women, a higher proportion of breakfast occasions characterised by “Protein-foods” were consumed after 9:00 h, and on the weekend, when compared to the other breakfast profiles. Irrespective of the breakfast profile, breakfast occasions tended to occur later on the weekend, than on weekdays, and a higher percentage of men, compared to women, reported a breakfast occasion before 7:00 h.

**Table 4:** Percentage of breakfast food profiles consumed according to the type of day (weekend vs. weekday) and time-period of the day among Australian men and women breakfast consumers^a, b, c^

	All breakfasts	Wholegrain cereals & milks	Protein-foods	Bread & spreads 1	Mixed cereals & milks	Bread & spreads 2	***P*** value
		***Men (n = 3545)***					
*Day of the week (%)*							< 0.001
Monday to Friday (weekday)	77	81	67	75	80	75	
Saturday/Sunday (weekend)	23	19	33	25	20	25	
*Weekday time-period (%)*							< 0.001
< 7:00	26	30	15	23	31	21	
7:00 to 7:59	30	36	20	40	28	22	
8:00 to 8:59	23	21	25	21	23	24	
≥ 9:00	21	13	40	16	18	33	
*Weekend time-period (%)*							0.08
< 7:00	12	18	6	11	14	8	
7:00 to 7:59	19	24	14	22	18	19	
8:00 to 8:59	26	26	21	30	28	24	
≥ 9:00	43	32	59	37	40	49	
		***Women (n = 4127)***					
*Day of the week (%)*							< 0.001
Monday to Friday	80	82	70	84	77	80	
Saturday/Sunday	20	18	30	16	23	23	
*Weekday time-period (%)*							< 0.001
< 7:00	15	16	9	16	16	14	
7:00 to 7:59	34	42	22	34	34	32	
8:00 to 8:59	28	27	30	28	28	30	
≥ 9:00	23	14	39	22	22	24	
*Weekend time-period (%)*							< 0.001
< 7:00	8	8	2	8	12	7	
7:00 to 7:59	22	41	11	23	16	24	
8:00 to 8:59	33	27	27	36	39	32	
≥ 9:00	37	24	60	33	33	37	

^a^Values are weighted percentages

^b^Weekday vs weekend and time-of-day differences between breakfast profiles for were assessed using an adjusted Pearson Chi2 test

^c^The Breads and spreads profiles were differentiated by their accompanying beverages for men (Profile 1: coffee/tea; Profile 2: Sugar-sweetened beverages) and by bread grain varieties for women (Profile 1: wholegrain; Profile 2: refined grain)

### Participant characteristics of latent breakfast food profiles

Table 5 presents the characteristics of Australian men and women, overall and by latent breakfast food profile. The mean ages of men and women were 47.0 y and 48.5 y, respectively. Over two thirds of men and women were born in Australia and were more likely to have a medium education level, live in major Australian cities and be married or in a defacto relationship. Compared with the other profiles, men with a “Wholegrain cereals and milks” or “Bread and Spreads 1” profile were older and a higher proportion were married*.* Men with a “Wholegrain cereals and milks” profile had higher education and income levels, a lower proportion were current smokers and a higher proportion were born in Australia, whereas men with a “Breads and spreads 1” profile had lower education and income levels, lived in an inner regional/rural city, and a higher proportion did not work in the previous week. Among women, those with a “Protein-centred” profile were younger and a higher proportion were born in a non-English speaking country, unmarried and worked < 40 h in the previous week. Women with a “Wholegrain cereals and milks” or either of the “Bread and spreads” profiles were older and, as per men, women with a “Wholegrain cereals and milks profile” were more likely to be born in Australia, meet physical activity guidelines and less likely to be current smokers, however, no associations were found for education or income level. Women with the "Wholegrain cereals and milks" and "Breads and Spreads 1"  profiles were also more likely to be married.

**Table 5:** Sociodemographic and health characteristics of Australian men and women breakfast consumers and their associations with breakfast food profiles^*1 − 3*^

	All breakfast consumers	Wholegrain cereals & milks	Protein-foods	Bread & spreads 1	Mixed cereals & milks	Bread & spreads 2	***P*** value
		***Men (n = 3545)***					
Age (years, mean (95% confidence interval))	47.0 (46.6, 47.4)	49.6 (47.7, 51.5)^a^	42.7 (41.0, 44.4)^bd^	54.7 (53.2, 56.1)^c^	45.1 (43.9, 46.2)^d^	40.2 (38.5, 41.9)^be^	< 0.001
*Country of birth (%)*							< 0.001
Australia	68	74	61	69	69	65	
Mainly English speaking	12	13	10	13	13	8	
All other countries	20	12	29	18	18	27	
*Education level (%)*							< 0.05
Low	22	17	20	26	23	23	
Medium	53	51	55	53	55	53	
High	25	32	25	21	22	25	
*Weekly income (quintiles, %)*^*4*^							< 0.05
First: <$398	16	13	13	21	17	16	
Second: $399–638	16	13	19	19	15	13	
Third: $639–958	21	19	21	20	20	24	
Fourth: $959–1438	23	27	22	20	24	24	
Fifth: ≥ $1439	24	28	25	21	24	22	
*Geographic region (%)*							< 0.001
Major cities of Australia	70	73	73	63	71	74	
Inner regional cities	20	17	15	25	20	19	
Other areas (rural/remote)	10	10	12	12	9	8	
Social status (%)							< 0.001
Married	64	71	58	72	60	57	
Not married	36	29	42	28	41	43	
Hours worked (past week, %)							< 0.001
None	33	31	29	45	31	23	
< 40 h	27	28	26	22	27	32	
≥ 40 h	41	41	44	33	43	45	
Smoking status (%)							< 0.001
Current smoker	16	10	26	14	16	19	
Former smoker	37	38	36	46	34	29	
Never smoker	47	52	39	40	49	52	
Met PA guidelines (%)^*5*^	45	51	43	42	42	50	< 0.05
		***Women (n = 4127)***					
Age (y, mean (95% confidence interval))	48.5 (48.1, 48.9)	52.1 (50.1, 54.1)^a^	40.9 (38.8, 43.0)^b^	52.0 (50.0, 54.1)^a^	47.1 (46.2, 48.1)^c^	49.1 (47.2, 50.9)^ac^	< 0.001
Country of birth (%)							< 0.001
Australia	70	76	60	70	70	69	
Mainly English speaking	11	13	8	12	12	9	
All other countries	19	11	32	18	18	22	
Education level (%)							0.09
Low	29	30	21	30	28	33	
Medium	43	42	51	44	42	42	
High	28	28	28	26	30	25	
Weekly income (quintiles, %)^*4*^							0.06
First: <$398	21	17	24	22	21	23	
Second: $399–638	19	21	13	22	19	21	
Third: $639–958	19	18	20	20	18	22	
Fourth: $959–1438	21	23	21	21	21	20	
Fifth: ≥ $1439	19	22	23	16	21	14	
Geographic region (%)							0.46
Major cities of Australia	73	73	78	69	72	73	
Inner regional cities	18	19	13	21	18	18	
Other areas (rural/remote)	9	8	9	10	9	9	
Social status (%)							< 0.01
Married/defacto	58	62	51	59	56	65	
Not married	42	38	49	41	44	35	
Hours worked (past week, %)							< 0.01
None	46	47	40	51	43	49	
< 40 h	37	38	43	33	37	38	
≥ 40 h	17	15	17	16	20	14	
Smoking status (%)							< 0.05
Current smoker	13	9	14	15	13	13	
Former smoker	28	32	25	30	26	27	
Never smoker	59	59	61	55	61	60	
Met PA guidelines (%)^5^	42	49	44	43	42	36	< 0.05

^*1*^Values are weighted means (95% confidence interval) for continuous variables and weighted proportions for categorical variables

^*2*^Different superscript letters indicate significant differences between profiles, assessed using a *F*-test with Bonferroni correction for continuous variables. Differences between profiles for categorical variables were assessed using an adjusted Pearson Chi2 test

^3^The Breads and spreads profiles were differentiated by their accompanying beverages for men (Profile 1: coffee/tea; Profile 2: Sugar-sweetened beverages) and by bread grain varieties for women (Profile 1: wholegrain; Profile 2: refined grain)

^*4*^*n* = 2258 men and *n* = 2586 women due to missing cases for income

^5^Whether met physical activity (PA) guidelines of 150 min and 5 sessions per week

### Adiposity characteristics of latent breakfast food profiles

Differences in adjusted adiposity estimates by latent breakfast food profile are presented in Table 6. Both men and women with a “Wholegrain cereals and milks” profile had the most favourable estimates for BMI and WC and overweight/obesity prevalence, when compared to the other profiles. However, among men with a “Wholegrain cereals and milks” profile, BMI and WC were only statistically significantly lower than men with either of the “Breads and spreads” (1 or 2) profile. Women with a “Wholegrain cereals and milks” had lower BMI and WC when compared to the “Mixed cereals and milks” profile and also a lower WC when compared to the “Protein-foods” and “Breads and Spreads 2” profiles. No statistically significant differences between breakfast profiles were found for the prevalence of overweight/obesity among either men or women.

**Table 6:** Adiposity characteristics of Australian men and women breakfast consumers and their associations with breakfast food profiles^*1 − 3*^

	All breakfast consumers	Wholegrain cereals & milks	Protein-foods	Bread & spreads 1	Mixed cereals & milks	Bread & spreads 2
	***Men***^***5***^					
BMI^*4*^, kg/m^2^	27.2 (27.0, 27.4)	26.6 (26.2, 27.1)^a^	27.1 (26.6, 27.7)	27.8 (27.3, 28.2)^b^	27.0 (26.6, 27.3)^a^	27.8 (27.2, 28.4)^b^
BMI ≥25 kg/m^2^, %	71 (68.6, 73.6)	68.9 (62.9, 74.8)	69.5 (63.7, 75.3)	75.0 (70.4, 79.4)	69.9 (61.1, 73.6)	71.6 (65.0, 78.1)
Waist circumference, cm	97.9 (97.3, 98.5)	95.4 (94.1, 96.8)^a^	97.8 (96.2, 99.4)^b^	99.4 (98.2, 100.5)^b^	97.2 (96.1, 98.3)^a^	99.9 (98.2, 101.5)^b^
Waist circumference, cm ≥94 cm, %	61.6 (58.7, 64.5)	58.3 (51.7, 64.9)	63.1 (56.1, 70.2)	66.4 (61.4, 71.4)^b^	58.5 (53.3, 63.8)	63.3 (56.7, 70.0)
	***Women***^***6***^					
BMI^*4*^, kg/m^2^	26.4 (26.1, 26.6)	25.6 (25.1, 26.1)^a^	26.3 (25.1, 27.0)	26.3 (25.7, 26.9)	26.9 (26.4, 27.4)^b^	26.2 (25.6, 26.8)
BMI ≥25 kg/m^2^, %	56.2 (53.2, 59.2)	50.7 (43.9, 57.6)	56.1 (48.0, 64.2)	58.4 (52.6, 64.2)	58.9 (54.3, 63.5)^b^	53.9 (48.0, 59.8)
Waist circumference, cm	87.5 (86.8, 88.3)	85.5 (84.1, 86.9)^a^	88.1 (86.3, 90.0)^b^	87.6 (86.0, 89.1)	88.3 (87.1, 89.5)^b^	87.5 (86.1, 89.0)^b^
Waist circumference, cm ≥80 cm, %	68.5 (65.9, 71.1)	64.6 (57.8, 71.5)	71.9 (64.8, 78.9)	69.0 (64.1, 74.0)	69.2 (65.0, 73.4)	68.1 (62.9, 73.1)

^1^Values are weighted means (95% confidence intervals) for continuous variables and weighted percentages (95% confidence intervals) for categorical variables adjusted for age, education level, smoking status, meeting physical activity guidelines and energy intake at all eating occasions outside of breakfast

^2^Different superscript letters indicate significant pairwise differences between breakfast profiles after applying the Bonferroni correction (*p* < 0.05)

^3^The Breads and spreads profiles were differentiated by their accompanying beverages for men (Profile 1: coffee/tea; Profile 2: Sugar-sweetened beverages) and by bread grain varieties for women (Profile 1: wholegrain; Profile 2: refined grain)

^4^Values for BMI (continuous variable) are weighted geometric means (95% confidence interval)

^5^*n* = 3061 with no missing data for BMI and *n* = 3068 with no missing data for waist circumference

^6^*n* = 3431 with no missing data for BMI and *n* = 3414 with no missing data for waist circumference

## Discussion

Using a LVMM approach, this study explored the combinations of foods eaten at the breakfast eating occasion in a nationally representative sample of Australian men and women. This is one the few studies among adults to use a data-driven method to understand how foods are consumed in relation to each other at eating occasions, and highlights breakfast-specific differences in diet quality. Five distinct breakfast food profiles were found among men and women, and these profiles varied by time of consumption, participant characteristics and adiposity indicators.

Our finding of five unique breakfast food profiles that were associated with timing of consumption, participant characteristics and adiposity measures underscores the usefulness of LVMM to objectively capture the permutations of food combinations at eating occasions. Exploratory and data-driven approaches are useful because they do not rely on a set of arbitrary criteria or researcher-driven assumptions to understand how people combine foods together to make a meal (i.e. meal patterns).

Few studies have examined food profiles at specific eating occasions using a data-driven approach [17–19]. Applying supervised data mining techniques to 51 food groups, Hearty and Gibney found that common food combinations at breakfast were: bread with eggs/meat products; bread and breakfast cereal; and bread or breakfast cereal (and/or both) together with fruit/juice; bread and breakfast among Irish adults [17]. In another national study of Irish adults, Woolhead et al. used frequent-sets data mining methods to identify common food combinations, based on 20 food groups, to aggregate dietary data into generic meals at eating occasions [18]. Most frequent combinations for the breakfast eating occasion included cereals and milk, often paired with bread, fruit and/or juice, and breads with “cooked breakfast” (not further defined), egg, fruit or juice. Schwedhelm et al. used Gaussian graphical models to understand the interrelations between foods at meals, including breakfast, in German adults [19]. Cereals were found to link strongly together with nuts and links with fresh fruits and legumes were also found. Two profiles were observed where bread was the central food, consumed together with spreads (i.e. margarine, butter and sugar & confectionery) or with processed meat and cheese. Milk/dairy foods and eggs were not assigned to a profile but were linked with cereals and bread, respectively. Vegetables also featured and linked together with meat (either processed or lean) and sauces [19]. While direct comparisons between these studies and the present study are limited due to differences in the number and type of food group input variables and analytic methods used; there are some shared characteristics of the observed food combinations, including cereals with fruit [17–19] and nuts [19], breads with spreads [19], eggs [17, 19] and processed meat [17, 19] and vegetables with meat [19]. However, these breakfast combinations are based on dietary data collected in Western populations and breakfast food profiles are likely to differ across cultures [16, 44, 45]; country-specific studies are needed for the development of meal-specific advice that reflects social, cultural and dietary preferences.

In the present study, sex differences and weekend vs weekday differences in the timing of the breakfast occasion were observed and may be indicative of different work routines and schedules [46]. Further, a breakfast food profile characterised by “Protein-foods” occurred more frequently on weekends and later in the morning (irrespective of the day of the week), when compared to the other food profiles. As protein-foods such meat and eggs may require more preparation and cooking time than cereals or breads, this suggests that time-availability may be an important factor in determining food intakes at breakfast. Whilst in the present study, women with a “Protein-foods profiles” were more likely to work less than 40 h a week and be unmarried. Contextual information regarding location of eating, meal preparation, sharing of meals and perception of time-availability are needed to better understand the factors that influence food choices and diet quality at eating occasions [47, 48].

Studies investigating the participant characteristics of breakfast food profiles among adults are rare and have compared breakfasts comprising “ready-to-eat” cereals versus “all other” breakfasts [49] or different breakfasts patterns based on common foods [50] in nationally-representative samples of adults from the U.S. In all of these studies, consumption of breakfast cereals was positively associated with indicators of ethnicity (white [49, 50], speaking English at home [49]) and socioeconomic position (food security [49], income [50] and education [50]). Age was also positively linked to breakfast cereal consumption in one study [49]. While these finding are difficult to compare to the present study which provided a detailed examination of food combinations at breakfast, some similarities are observed. For example, we found that adults with a “Wholegrain cereals and milks” profile tended to be older and born in Australia, and among men, more highly educated. Compared to other breakfast types, Siega-Riz et al. found that breakfasts comprising egg as the main food were associated with being black or Hispanic and lower education, when compared to other breakfast types [50]. In the present study, both men and women with a “Protein-foods” profile (which included eggs) were more likely to be younger and born in a non-English speaking country and men with a “Bread and spreads 1” (which included tea/coffee) profile were older and had lower education and income and were more likely to live regionally. In contrast to our study, Barr et al. found no association between breakfast cereal consumption and marital status [49]. However, the present study examined wholegrain cereals and refined cereals as separate food groups, and in combination with other (more or less nutritious) foods, which may explain the different findings. Nonetheless, overall these results indicate that the sociocultural influences on food choices at breakfast are complex and may differ for men and women. Further research, including those with qualitative study designs, is needed to elucidate the factors that influence food choices and preferences so that approaches to improve breakfast diet quality can be tailored more effectively.

A wealth of research has focused on the health impacts of breakfast skipping, particularly weight-related outcomes with fewer studies examining the types and combinations of foods consumed at breakfast [35, 49, 51]. In the present study, men and women with a “Wholegrain cereals and milks” profile, which featured fruit, dried fruit, nuts and seeds and/or yoghurt, consistently had the lowest adiposity measures. This finding is in line with previous research that showed North American adults who consumed ready-to-eat cereals [33, 49] and breakfasts that included grains/cereals, reduced fat milks and/or fruit [35] had a more favourable BMI [33, 35] and WC [33, 35]. While the present cross-sectional analysis adjusted for energy intake and a number of important lifestyle confounders, a causal link between breakfast consumption and obesity has not been established [52]. Both breakfast consumption and breakfast quality are associated with other lifestyle factors such as better overall diet quality [7, 17, 35, 51], higher physical activity [49, 53] and non-smoking status [49, 53, 54]. In the present study, men and women with the “Wholegrain cereals and milks” profile had the lowest representation of current smokers and the highest representation meeting national physical activity guidelines. Further, a recent gene-wide association study using the UK Biobank data reported genetic links between skipping breakfast (defined as not eating any breakfast cereal on the previous day) and a higher BMI, more depressive symptoms and smoking status [55]. In the same study, genetically defined evening chronotype (i.e., being an evening person) predicted breakfast skipping. Thus, it is possible that the breakfast’s contribution to health is, in part due to, its association with an overall healthier lifestyle. The link between circadian clock regulation and habitual breakfast consumption, and in the context of sleep-wake and meal timing pattens, also merits investigation in future longitudinal studies.

A limitation of the present study is the breakfast food profiles were determined using a data-driven method that did not allow for the use of survey replicate weights and may limit the generalizability of findings beyond the study sample, including populations from other countries. In addition, little is known about the accuracy of self-reported eating patterns. Dietary intake at breakfast in this study was assessed using a single 24-h dietary recall which is prone to measurement error (e.g., portion size estimation, recall bias and underreporting of food intakes) and is unlikely to capture day-to-day variability in breakfast food profiles [56, 57]. Whilst the dietary recall varied by day of the week and allowed comparison of breakfast profiles according to weekday and weekend days, Sundays were underrepresented, and thus dietary intakes may not be representative of weekend intakes [57]. Further investigation is needed to understand how breakfast food profiles vary from day-to-day, including shorter term variations (e.g., weekend/weekday patterns, seasonal patterns) and longer term, across life-stages. Breakfast was also classified based on participant-identification of eating occasions, and not the time-of-day, which involves subjectivity in participants’ allocation of breakfast as their first eating occasion of the day. The researcher must also decide how to categorise eating occasions that are not clearly defined as breakfast (e.g., brunch), but are often treated as breakfast in studies because they are the first eating occasion of the day. Another subjective aspect is the categorisation of foods and beverages prior to analysis and is considered an impediment to comparing results across studies of dietary patterns [58]. The present study also focussed only on foods consumed at the breakfast occasion; we did not examine the absence of breakfast or profiles of food combinations at other eating occasions across the day. The cross-sectional design of this study also limits our ability to infer any causal relationships between breakfast profiles and adiposity. Further longitudinal investigation of different eating occasions, including their associations with sociodemographic and health indicators, is warranted.

A key strength of the present study was the objective LVMM approach to capture the variability of possible food combinations at breakfast eating occasions using nationally representative dietary data that reflects population intakes of Australian men and women aged 19 years and over. The present analysis examined population food group intakes at breakfast using a food classification scheme developed by the ABS that aligned with Australia’s current Dietary Guidelines. The identified breakfast profiles can be used to provide insight into what foods could be added or replaced at the breakfast eating occasions (e.g., targeted and meal-specific dietary advice) in order to recommend feasible dietary changes within particular styles of eating and improve the nutritional quality of the diet. For example, meal-specific messages that encourage the replacement of refined-grain cereals or breads with wholegrain varieties [59], processed meats with unprocessed meats [60] and the addition of vegetables (to quality protein foods) or nuts, fruit and yoghurts (to cereals) are other examples where breakfast quality could be improved. The person-centred approach of LVMM also provides useful information about sub-populations with suboptimal intake of specific foods at eating occasions and who could be targeted as part of public health strategies. For example, older Australian men who live in regional centres and consume mainly breads and spreads at breakfast may be an important group to target with messages to increase intake of protein rich foods as part of broader strategies to promote healthy aging [61]. Finally, meal and eating occasion profiles may inform modelling of dietary patterns in the translation of dietary guideline and nutrient reference values when developing food guides and other public health nutrition tools through the understanding of consumer behaviour and preferences [62, 63].

In conclusion, LVMM was a novel and useful approach for capturing the complexity of food combinations at eating occasions. Five distinct breakfast food profiles that varied by time of consumption, participant characteristics and adiposity measures were identified among a representative sample of Australian men and women. Understanding how foods are combined together to form eating occasions will inform the development of targeted nutritional strategies that aim to improve diet quality. However, future research is needed to understand the sociocultural and contextual influences on food choices at eating occasions and to examine food profiles at other eating occasions across the day (e.g. lunch, dinner, and snacks) in relation to health markers.

## Supplementary Information

**Additional file 1.** Participant flowchart for inclusion in the analysis of breakfast food profiles and their associations with food intake, participant characteristic and adiposity measures.

**Additional file 2.** Food groups at breakfast eating occasions used as input variables for the latent variable mixture modelling.

**Additional file 3 **Reported consumption (%) of food group intake at the breakfast eating occasion among Australian men who completed the second recall day (*n*=2320), by latent breakfast profile.

**Additional file 4 **Reported consumption (%) of food group intake at the breakfast eating occasions among Australian women who completed the second recall day (*n*=2770), by latent breakfast profile.

**Additional file 5 **Energy-adjusted food group intakes at the breakfast eating occasion according to breakfast profile among Australian men breakfast consumers (*n*=3545).

**Additional file 6 **Energy-adjusted food group intakes at the breakfast eating occasion according to breakfast profile among Australian women breakfast consumers (*n*=4127).

## Data Availability

Data are available on request from the Australian Bureau of Statistics from https://www.abs.gov.au/websitedbs/D3310114.nsf/home/Microdata%2BEntry%2BPage. The research team does not have the permission to release the data to third parties.

## Abbreviations

ABS: Australian Bureau of Statistics
ADG: Australian Dietary Guidelines
AUSNUT: Australian Food and Nutrient Database
BMI: Body mass index
CI: Confidence interval
LVMM: Latent variable mixture modelling
OR: Odds ratio
RGLF: Refined grain or lower fibre
SSB: Sugar-sweetened beverages
WC: Waist circumference
WGHF: Wholegrain or high fibre
