# Supplementary material for: What do Australian adults eat for breakfast? A latent variable mixture modelling approach for understanding combinations of foods at eating occasions
Source: Int J Behav Nutr Phys Act. 2021 Mar 25;18:46. doi: 10.1186/s12966-021-01115-w (PMC7992839; doi:10.1186/s12966-021-01115-w)
Supplement: Supplementary file 1 — Additional file 1. Participant flowchart for inclusion in the analysis of breakfast food profiles and their associations with food intake, participant characteristic and adiposity measures. [file 12966_2021_1115_MOESM1_ESM.docx]

**Additional File 1.** Participant flowchart for inclusion in the analysis of breakfast food profiles and their associations with food intake, participant characteristic and adiposity measures
