# Supplementary material for: What do Australian adults eat for breakfast? A latent variable mixture modelling approach for understanding combinations of foods at eating occasions
Source: Int J Behav Nutr Phys Act. 2021 Mar 25;18:46. doi: 10.1186/s12966-021-01115-w (PMC7992839; doi:10.1186/s12966-021-01115-w)
Supplement: Supplementary file 2 — Additional file 2. Food groups at breakfast eating occasions used as input variables for the latent variable mixture modelling. [file 12966_2021_1115_MOESM2_ESM.docx]

**Additional 2:** Food groups at breakfast eating occasions used as input variables for the latent variable mixture modelling

| **Food group label** | **Additional criteria used by the ABS to classify foods, where applicable** | **Some example foods within the food group category** |
| --- | --- | --- |
| *Non-discretionary food groups* | | |
| 1. Whole grain or high fibre breads | Contains >5g/100g dietary fibre (breads and crackers) and >10g/100g dietary fibre (crumpets, muffins and scones) | Breads/bread rolls; Crispbreads; Crackers; Savoury biscuits; Scones; Muffins; Crumpets; Pancakes, Pikelets or Crepes |
| 1. Whole grain or high fibre cereals | Contains ≥5g dietary fibre and <30g sugar (<35g if contains fruit) per 100g | Muesli (toasted or untoasted); wheat bran pellets, mixed grain breakfast cereals (i.e. wheat, corn or oat) breakfast cereal flakes or clusters, whole wheat breakfast biscuits |
| 1. Whole (high fibre) grains |  | Porridge, rolled oats, wholegrains or wholemeal flours (used in recipes/mixed dishes); wholemeal pasta, brown rice, rice bran |
| 1. Refined grain or lower fibre breads | White wheat flour based, or contains <10g/100g dietary fibre and <15g/100g total fat | Breads/bread rolls, crispbreads, crackers, scones, muffins, crumpets; pancakes, pikelets or crepes |
| 1. Refined grain or lower fibre cereals | Contains <5g dietary fibre and <30g sugar (<35g if contains fruit) per 100g | Mixed grain breakfast cereals (i.e. combination of wheat, corn, oat, rice); breakfast cereal flakes of corn or mixed grain; puffed or popped rice breakfast cereals |
| 1. Refined (lower fibre) grains |  | White rice; instant wheat noodles; white flour (used in recipes/mixed dishes), pasta, cornmeal (polenta), corn taco shells, couscous |
| 1. Green and Brassica Vegetables |  | Lettuce, spinach, rocket, mixed leafy greens, broccoli, broad beans, green peas, parsley, basil |
| 1. Orange vegetables |  | Carrot, pumpkin |
| 1. Starchy vegetables |  | Sweet potato, potato varieties (including peeled/unpeeled), corn, parsnip, taro |
| 1. Legumes (consumed as a vegetable or meat alternative) |  | Baked beans, tofu, mixed beans, dried/split peas, chickpeas, black beans, lentils |
| 1. All other vegetables |  | Tomato, tomato-based sauce/paste, vegetable juices, garlic, onion, celery, cucumber, capsicum, mushroom, zucchini |
| 1. Fruit – fresh or canned |  | Bananas, pears, melons, grapes, stone fruits, citrus fruits, pineapple, berries, apples |
| 1. Fruit – dried |  | Sultanas, prunes, raisins, dried apricots, mixed dried fruit |
| 1. Reduced fat milks   (including milk alternatives) | Contains <4% fat  Milk alternative contains at least 100mg calcium/100mL | Skim cow’s milk, reduced fat cow’s milk, soy milk, rice milk, reduced fat flavoured cow’s milk, reduced fat evaporated milk |
| 1. Medium fat milks | Contains 4-10% fat  Milk alternative contains at least 100mg calcium/100mL | Regular fat cow’s milk, regular fat flavoured cow’s milk, regular fat soy milk, regular fat evaporated milk |
| 1. Yoghurts and custards |  | Natural yoghurt, Greek yoghurt, flavoured yoghurt, yoghurt dressings, yoghurt desserts , flavoured custards, fromage frais |
| 1. Cheeses |  | Hard cheese (cheddar, parmesan, Colby, haloumi), soft cheese (ricotta, cottage, goat, brie), cream cheese |
| 1. Lean red meat | Contains <10% fat | Beef, lamb or goat (semi/fully-trimmed), kangaroo, pork |
| 1. Lean Poultry <10% fat | Contains <10% fat | Chicken, turkey, duck |
| 1. Fish |  | Salmon, barramundi, tuna, prawns, anchovies, squid, canned fish, shellfish |
| 1. Eggs |  | Chicken eggs, duck eggs |
| 1. Nuts and seeds |  | Nuts (almonds, cashews, mixed nuts), nuts in pastes/spreads, peanuts, seeds (linseed, sunflower), tahini |
| 1. Unsaturated spreads | Contains predominantly polyunsaturated and monounsaturated fatty acids | Margarine spread (monounsaturated,  polyunsaturated or olive oil blend) |
| 1. Unsaturated oils | Contains predominantly polyunsaturated and monounsaturated fatty acids | Olive oil, vegetable oil, linseed or flaxseed oil, nut oil |
| 1. Water |  | Water (tap, bottled, still, filtered, rainwater or tank water), Mineral water (natural, plain or unflavoured).  Bottled water (carbonated or soda) |
| 1. Fruit juices | Contains 100% fruit juice | Juice (orange, apple, tropical, pineapple), fruit juice blends (with vegetables) |
| 1. Teas and coffees |  | Tea (black, green, jasmine, teabag/loose leaf), coffee (black, espresso, instant, from ground beans |
| *Discretionary food groups* | | |
| 1. Processed meats |  | Bacon, sausage, salami, ham, deli-sliced turkey, kabana or cabanossi, corned beef, processed luncheon meat |
| 1. Sugar sweetened beverages |  | Flavoured soft drink (includes flavoured teas), flavoured mineral water, flavoured tonic water, fruit drink, cordials, energy drinks, sports drinks, flavoured beverage-bases |
| 1. Sweet cereal products |  | Muffins, banana bread, sweet pies, cakes, cupcakes, brownies, sweet biscuits/cookies, tarts, wafer cones, rumballs, doughnuts, sweet buns, puddings, lamingtons, slices |
| 1. Savoury cereal products |  | Savoury biscuits/crackers, sausage rolls, pasties, pies, quiche, croissants, dumplings/wontons, commercial garlic/herb breads, spring rolls |
| 1. Discretionary cereals | Ready to eat breakfast cereal contains <5g dietary fibre and >30g sugar (or >35g if contains fruit) per 100g  Snack/muesli bars: 73% of bar contains ≥20g sugar per 100g or 93% contains ≥15g (includes dried fruit) sugar per 100g | Puffed or popped rice, crispy (mixed grain) pillow breakfast cereal with cocoa coating; fruit flavoured extruded breakfast cereals, fruit filled baked muesli/snack bars, energy cereal/snack bars, muesli/snack bars with dried fruit, yoghurt/chocolate coated |
| 1. Sugar | Added by participants to recipes, foods, dishes | Sugar (white, granulated or lump, raw, fructose, brown, cinnamon) |
| 1. Condiments |  | Sauces (soy, tomato, barbecue), dressings, commercial curry/casserole pastes/bases, chutney or relish, mayonnaise, mustard, dips |
| 1. Discretionary spreads |  | Jam, butter/butter blends, marmalade, honey, yeast spread, hazelnut/chocolate flavoured spreads |

Abbreviations: ABS, Australian Bureau of Statistics
