# Supplementary material for: What do Australian adults eat for breakfast? A latent variable mixture modelling approach for understanding combinations of foods at eating occasions
Source: Int J Behav Nutr Phys Act. 2021 Mar 25;18:46. doi: 10.1186/s12966-021-01115-w (PMC7992839; doi:10.1186/s12966-021-01115-w)
Supplement: Supplementary file 3 — Additional file 3 Reported consumption (%) of food group intake at the breakfast eating occasion among Australian men who completed the second recall day (n=2320), by latent breakfast profile. [file 12966_2021_1115_MOESM3_ESM.docx]

**Additional File 3**: Reported consumption (%) of food group intake at the breakfast eating occasion among Australian men who completed the second recall day (n=2320), by latent breakfast profile^1^

| Food group (grams) |  | **WGHF cereals**  **& milks (13 %)** | **Protein-foods**  **(12 %)** | **Breads & spreads 1**  **(11 %)** | **Mixed cereals**  **& milks (39 %)** | **Breads & spreads 2 (26 %)** |
| --- | --- | --- | --- | --- | --- | --- |
| WGHF cereals |  | **97** | 1 | 68 | 34 | <1 |
| RGLF cereals |  | 1 | 0 | 14 | 15 | 2 |
| Discretionary cereals |  | 0 | 0 | <1 | **7** | 1 |
| WGHF breads |  | <1 | 23 | **57** | <1 | 45 |
| RGLF breads |  | 0 | **51** | 47 | 1 | **50** |
| WGHF grains |  | 14 | 13 | **21** | 16 | 3 |
| RGLF grains |  | 3 | **16** | 3 | 1 | <1 |
| Fresh/canned fruit |  | **36** | 9 | 29 | 19 | 10 |
| Dried fruit |  | **89** | 2 | 42 | 3 | 4 |
| Brassica vegetables |  | <1 | **26** | 1 | 0 | <1 |
| Orange vegetables |  | <1 | **13** | 0 | 0 | 0 |
| Starchy vegetables |  | <1 | **14** | 0 | 0 | <1 |
| Legumes |  | 2 | **6** | <1 | 0 | 5 |
| All other vegetables |  | 2 | **42** | 8 | <1 | 5 |
| Reduced fat milks |  | 40 | 6 | **41** | 30 | 18 |
| Medium fat milks |  | 44 | 28 | 49 | **50** | 27 |
| Yoghurts & custard |  | **22** | 3 | 8 | 3 | 3 |
| Cheeses |  | 0 | **27** | 1 | 0 | 10 |
| Lean red meat |  | <1 | **5** | <1 | 0 | 0 |
| Lean poultry |  | 0 | **6** | <1 | 0 | <1 |
| Fish |  | 0 | **8** | 0 | 0 | 2 |
| Processed meats |  | 0 | **42** | 2 | 0 | 7 |
| Eggs |  | 1 | **61** | 3 | <1 | 12 |
| Nuts & seeds |  | **44** | 0 | 21 | <1 | 8 |
| Unsaturated oils |  | 29 | **67** | 19 | <1 | 9 |
| Unsaturated spreads |  | 2 | 31 | **47** | 0 | 39 |
| Discretionary spreads |  | 6 | 10 | **73** | 9 | 50 |
| Condiments |  | 0 | **18** | 0 | <1 | 3 |
| Fruit juice (100%) |  | 14 | **19** | 15 | 8 | 8 |
| Water |  | 20 | 17 | 15 | 18 | **23** |
| Tea/coffee |  | 49 | 42 | **70** | 43 | 59 |
| Sugar |  | 16 | 13 | **37** | 31 | 31 |
| SSBs |  | 3 | **14** | 5 | 4 | 8 |
| Sweet cereal products |  | 0 | <1 | 3 | **4** | 3 |
| Savoury cereal products |  | 0 | <1 | 0 | **3** | 2 |

^1^Values shown are weighted percentage (%) of men who reported consuming one or more food/beverage items from each food group at breakfast. Values in bold indicate the highest proportion of consumption across breakfast profiles for each food group. Abbreviations: RGLF, refined grain or lower fibre; SSBs: sugar-sweetened beverages; WGHF, wholegrain or high fibre
