# Supplementary material for: What do Australian adults eat for breakfast? A latent variable mixture modelling approach for understanding combinations of foods at eating occasions
Source: Int J Behav Nutr Phys Act. 2021 Mar 25;18:46. doi: 10.1186/s12966-021-01115-w (PMC7992839; doi:10.1186/s12966-021-01115-w)
Supplement: Supplementary file 4 — Additional file 4 Reported consumption (%) of food group intake at the breakfast eating occasions among Australian women who completed the second recall day (n=2770), by latent breakfast profile. [file 12966_2021_1115_MOESM4_ESM.docx]

**Additional File 4**: Reported consumption (%) of food group intake at the breakfast eating occasions among Australian women who completed the second recall day (n=2770), by latent breakfast profile^1^

| Food group (grams) |  | **WGHF cereals**  **& milks (18 %)** | **Protein-foods**  **(13 %)** | **Breads & spreads 1**  **(15 %)** | **Mixed cereals & milks (38 %)** | **Breads & spreads 2**  **(16 %)** |
| --- | --- | --- | --- | --- | --- | --- |
| WGHF cereals |  | **98** | <1 | 8 | 26 | 6 |
| RGLF cereals |  | 5 | <1 | <1 | **13** | 3 |
| Discretionary cereals |  | <1 | 0 | <1 | **4** | <1 |
| WGHF breads |  | 10 | 14 | **99** | <1 | 8 |
| RGLF breads |  | 5 | 33 | 0 | <1 | **100** |
| WGHF grains |  | 12 | 16 | 9 | **30** | 3 |
| RGLF grains |  | <1 | **32** | 0 | 2 | <1 |
| Fresh/canned fruit |  | **41** | 13 | 14 | 28 | 11 |
| Dried fruit |  | **84** | 5 | 2 | 4 | 16 |
| Brassica vegetables |  | <1 | **15** | 0 | <1 | <1 |
| Orange vegetables |  | 0 | **6** | 0 | 0 | 0 |
| Starchy vegetables |  | <1 | **9** | 0 | 0 | 0 |
| Legumes |  | 3 | **8** | 3 | 1 | 1 |
| All other vegetables |  | 2 | **37** | 7 | <1 | 4 |
| Reduced fat milks |  | **59** | 14 | 39 | 38 | 20 |
| Medium fat milks |  | 26 | 24 | 23 | **37** | 31 |
| Yoghurts & custard |  | **26** | 5 | 4 | 11 | 2 |
| Cheeses |  | **<1** | **22** | 9 | <1 | 7 |
| Lean red meat |  | 0 | **3** | 0 | 0 | <1 |
| Lean poultry |  | 0 | **3** | 0 | <1 | 0 |
| Fish |  | 0 | **5** | <1 | 0 | 1 |
| Processed meats |  | 0 | **22** | 4 | <1 | 4 |
| Eggs |  | <1 | **50** | 11 | 2 | **8** |
| Nuts & seeds |  | **45** | 3 | 13 | 5 | 9 |
| Unsaturated oils |  | 30 | **62** | 14 | <1 | 9 |
| Unsaturated spreads |  | 5 | 25 | 35 | 0 | **39** |
| Discretionary spreads |  | 12 | 13 | **60** | 5 | **60** |
| Condiments |  | <1 | **6** | 3 | 1 | 2 |
| Fruit juice (100%) |  | 7 | **10** | 7 | 7 | 6 |
| Water |  | **27** | 26 | 18 | 23 | 18 |
| Tea/coffee |  | 54 | 38 | 76 | 49 | 66 |
| Sugar |  | 13 | 9 | 26 | 26 | **27** |
| SSBs |  | <1 | **5** | 4 | **5** | 4 |
| Sweet cereal products |  | <1 | 2 | 3 | **4** | <1 |
| Savoury cereal products |  | <1 | **3** | <1 | 2 | 0 |

^1^Values shown are weighted percentage (%) of men who reported consuming one or more food/beverage items from each food group at breakfast. Values in bold indicate the highest proportion of consumption across breakfast profiles for each food group. Abbreviations: RGLF, refined grain or lower fibre; SSBs: sugar-sweetened beverages; WGHF, wholegrain or high fibre
