# Supplementary material for: What do Australian adults eat for breakfast? A latent variable mixture modelling approach for understanding combinations of foods at eating occasions
Source: Int J Behav Nutr Phys Act. 2021 Mar 25;18:46. doi: 10.1186/s12966-021-01115-w (PMC7992839; doi:10.1186/s12966-021-01115-w)
Supplement: Supplementary file 5 — Additional file 5 Energy-adjusted food group intakes at the breakfast eating occasion according to breakfast profile among Australian men breakfast consumers (n=3545). [file 12966_2021_1115_MOESM5_ESM.docx]

**Additional File 5.** Energy-adjusted food group intakes at the breakfast eating occasion according to breakfast profile among Australian men breakfast consumers (n=3545).^1^

| Food group (grams) | **All breakfasts** | | **Wholegrain cereals**  **& milks (16%)** | | **Protein-foods (11%)** | | **Bread & spreads 1 (23%)** | | **Mixed cereals & milks (33%)** | | **Bread & spreads 2 (15%)** | |
| --- | --- | --- | --- | --- | --- | --- | --- | --- | --- | --- | --- | --- |
|  | *%* | *Geometric mean*  *(95% CI)* | *%* | *Geometric mean*  *(95% CI)* | *%* | *Geometric mean*  *(95% CI)* | *%* | *Geometric mean*  *(95% CI)* | *%* | *Geometric mean*  *(95% CI)* | *%* | *Geometric mean*  *(95% CI)* |
| WGHF cereals | 33 | 40.0  (38.3, 41.7) | **99** | 38.4  (36.3, 40.6) | 3 | - | 16 | 26.2  (23.6, 29.1) | 38 | 48.6  (46.2, 51.2) | 0 | **-** |
| RGLF cereals | 8 | 31.9  (29.2, 34.8) | 5 | 16.9  (11.7, 24.3) | 0 | - | 6 | 26.4  (21.4, 32.6) | **17** | 36.0  (32.9, 39.4) | 2 | - |
| Discretionary cereals | 3 | 44.3  (37.9, 51.8) | <1 | - | <1 | - | 1 | - | **6** | 48.4  (42.0, 55.9) | 2 | - |
| WGHF breads | 20 | 55.1  (52.3, 58.1) | 9 | 40.7  (35.6, 46.5) | 23 | 41.8  (35.4, 49.4) | **46** | 55.7  (52.3, 59.2) | 0 | - | 34 | 67.6  (62.0, 73.8) |
| RGLF breads | 25 | 57.0  (54.0, 60.1) | 7 | 35.9  (30.0, 42.9) | 49 | 49.7  (43.8, 56.5) | **51** | 59.1  (54.8, 63.8) | 0 | - | 41 | 65.5  (61.0, 70.4) |
| WGHF grains | 12 | 93.9  (76.7, 114.9) | 15 | 20.3  (11.7, 35.4) | 7 | 52.0  (28.9, 93.6) | 10 | 148.1  (117.6, 186.6) | **17** | 191.0  (169.5, 215.3) | 5 | 25.4  (10.8, 59.6) |
| RGLF grains | 4 | 14.4  (8.7, 23.8) | <1 | - | **15** | 17.5  (8.0, 38.6) | 1 | - | <1 | - | 8 | 13.7  (7.4, 25.7) |
| Fresh/canned fruit | 18 | 72.9  (63.4, 83.9) | **37** | 41.9  (31.9, 55.1) | 6 | 54.6  (24.0, 124.3) | 13 | 97.7  (77.6, 123.0) | 19 | 100.3  (80.2, 125.4) | 9 | 113.1  (70.6, 181.2) |
| Dried fruit | 18 | 10.8  (9.9, 11.8) | **87** | 10.0  (9.2, 11.0) | <1 | - | 10 | 10.2  (8.5, 12.3) | 3 | - | 4 | - |
| Brassica vegetables | 4 | 12.6  (9.8, 16.2) | <1 | - | **33** | 13.6  (10.9, 16.9) | 1 | - | <1 | - | 0 | - |
| Orange vegetables | 2 | 11.8  (8.5, 16.4) | 0 | - | **14** | 11.4  (8.4, 15.5) | 0 | - | <1 | - | 0 | - |
| Starchy vegetables | 2 | 14.4  (8.7, 23.6) | 0 | - | **13** | 12.0  (7.6, 18.9) | <1 | - | 0 | - | 1 | - |
| Legumes | 2 | 74.0  (42.9, 127.6) | <1 | - | **7** | 12.7  (4.2, 38.0) | 3 | 166.1  (114.9, 239.9) | <1 | - | 4 | 289.5  (148.9, 563.0) |
| All other vegetables | 9 | 43.4  (35.5, 53.0) | 2 | - | **51** | 34.5  (28.5, 41.7) | 6 | 67.4  (44.0,103.4) | <1 | - | 10 | 53.4  (24.5, 116.4) |
| Reduced fat milks | 27 | 110.8  (101.8, 120.6) | **51** | 127.3  (112.8, 143.8) | 8 | 40.9  (30.8, 54.2) | 28 | 53.6  (46.4, 62.0) | 32 | 159.2  (140.3, 180.7) | 5 | 229.2  (194.2, 270.7) |
| Medium fat milks | 39 | 96.8  (90.5, 103.5) | 38 | 106.1  (93.4, 120.5) | 28 | 39.0  (32.5, 46.7) | 46 | 52.1  (46.3, 58.5) | **50** | 154.6  (143.6, 166.5) | 11 | 183.6  (140.2, 240.4) |
| Yoghurts & custard | 6 | 78.9  (71.5, 87.0) | **20** | 64.8  (57.1, 73.5) | <1 | - | 4 | - | 6 | 108.1  (88.2, 132.4) | 2 | - |
| Cheeses | 6 | 20.5  (18.0, 23.4) | 0 | - | **25** | 16.6  (13.7, 20.1) | 7 | 22.3  (17.1, 29.2) | <1 | - | 10 | 27.4  (22.5, 33.3) |
| Lean red meat | 2 | 55.1  (44.2, 68.6) | 0 | - | **12** | 55.9  (43.3, 72.2) | <1 | - | 0 | - | 1 | - |
| Lean poultry | 1 | 49.5  (39.4, 62.3) | 0 | - | **9** | 47.2  (39.1, 56.9) | <1 | - | 0 | - | 1 | - |
| Fish | 1 | 21.6  (8.5, 55.1) | 0 | - | **8** | 12.2  (4.1, 36.0) | <1 | - | 0 | - | 2 | - |
| Processed meats | 8 | 35.4  (31.9, 39.4) | 0 | - | **43** | 35.7  (32.0, 39.8) | 6 | 37.9  (30.0, 47.9) | <1 | - | 11 | 36.3  (27.9, 47.2) |
| Eggs | 13 | 63.3  (58.5, 68.6) | <1 | - | **61** | 54.9  (48.2, 62.6) | 15 | 73.2  (65.5, 81.9) | <1 | - | 15 | 82.2  (69.6, 97.0) |
| Nuts & seeds | 11 | 6.5  (5.8, 7.2) | **45** | 5.3  (4.7, 5.9) | 2 | - | 9 | 10.6  (7.9, 14.3) | 2 | - | 7 | 11.4  (8.8, 14.8) |
| Unsaturated oils | 18 | 2.9  (2.7, 3.1) | 30 | 2.1  (2.0, 2.2) | **69** | 3.9  (3.5, 4.3) | 11 | 2.3  (2.0, 2.7) | <1 | - | 17 | 2.8  (2.3, 3.3) |
| Unsaturated spreads | 20 | 5.9  (5.4, 6.5) | 5 | 4.9  (3.7, 6.5) | 43 | 1.9  (1.6, 2.1) | **44** | 9.0  (8.3, 9.7) | 0 | - | 25 | 8.8  (7.2, 10.7) |
| Discretionary spreads | 28 | 11.8  (11.1, 12.7) | 18 | 8.9  (7.4, 10.7) | 8 | 7.2  (5.9, 8.9) | **65** | 12.2  (11.1, 13.4) | 9 | 19.2  (16.6, 22.2) | 39 | 10.6  (8.9, 12.7) |
| Condiments | 4 | 14.5  (12.7, 16.6) | 0 | - | **20** | 16.4  (13.7, 19.5) | 2 | 11.1  (9.0, 13.6) | 0 | - | 7 | 12.3  (9.5, 15.8) |
| Fruit juice (100%) | 10 | 222.0  (198.1, 248.7) | **17** | 210.1  (181.0, 243.8) | 11 | 234.5  (151.8, 362.5) | 9 | 163.4  (124.8, 214.0) | 6 | 236.8  (206.7, 271.3) | 14 | 292.2  (216.7, 394.0) |
| Water | 20 | 712.2  (627.1,808.9) | **24** | 574.2  (460.8, 715.4) | 21 | 687.1  (503.2, 938.2) | 16 | 637.5  (481.3, 844.2) | 19 | 692.4  (564.2, 849.6) | **24** | 1080.2  (792.0, 1473.2) |
| Tea/coffee | 50 | 221.2  (212.6,230.1) | 51 | 206.9  (186.9, 229.2) | 50 | 235.9  (217.4, 256.0) | **90** | 205.6  (192.9, 219.1) | 44 | 236.6  (221.9, 252.3) | 2 | - |
| Sugar | 30 | 8.7  (8.3, 9.1) | 19 | 7.2  (6.2, 8.5) | 28 | 6.9  (5.9, 8.1) | **50** | 7.7  (7.2, 8.4) | 37 | 10.8  (10.0, 11.6) | 0 | - |
| SSBs | 7 | 107.4  (81.4, 141.6) | 3 | - | 12 | 157.1  (99.4, 248.3) | 4 | - | 5 | 51.7  (25.8, 103.7) | **16** | 167.7  (110.1, 255.5) |
| Sweet cereal products | 2 | 45.9  (37.2, 56.6) | <1 | - | <1 | - | 1 | - | **6** | 60.1  (53.4, 67.6) | <1 | - |
| Savoury cereal products | 2 | 65.0  (51.2, 82.5) | <1 | - | 4 | - | 1 | - | 2 | - | **6** | 92.5  (67.2, 127.5) |

^1^Values shown are weighted percentage (%) of women who reported consuming one or more food/beverage items from each food group at the breakfast eating occasion and weighted geometric mean (95% confidence interval) intake of food groups (where percentage of consumption by latent breakfast profile is ≥ 5%). Values in bold indicate the highest proportion of consumption across breakfast profiles for each food group. Abbreviations: RGLF, refined grain or lower fibre; SSBs: sugar-sweetened beverages; WGHF, wholegrain or high fibre
