# Supplementary material for: What do Australian adults eat for breakfast? A latent variable mixture modelling approach for understanding combinations of foods at eating occasions
Source: Int J Behav Nutr Phys Act. 2021 Mar 25;18:46. doi: 10.1186/s12966-021-01115-w (PMC7992839; doi:10.1186/s12966-021-01115-w)
Supplement: Supplementary file 6 — Additional file 6 Energy-adjusted food group intakes at the breakfast eating occasion according to breakfast profile among Australian women breakfast consumers (n=4127). [file 12966_2021_1115_MOESM6_ESM.docx]

**Additional File 6.** Energy-adjusted food group intakes at the breakfast eating occasion according to breakfast profile among Australian women breakfast consumers (n=4127)^1^

| Food group (g) | **All breakfasts** | | **Wholegrain cereals**  **& milks (17%)** | | **Protein-foods (11%)** | | **Bread & spreads 1 (18%)** | | **Mixed cereals & milks (37%)** | | **Bread & spreads 2 (17%)** | |
| --- | --- | --- | --- | --- | --- | --- | --- | --- | --- | --- | --- | --- |
|  | *%* | *Geometric mean*  *(95% CI)* | *%* | *Geometric mean*  *(95% CI)* | *%* | *Geometric mean*  *(95% CI)* | *%* | *Geometric mean*  *(95% CI)* | *%* | *Geometric mean*  *(95% CI)* | *%* | *Geometric mean*  *(95% CI)* |
| WGHF cereals | 29 | 36.2  (34.8, 37.8) | **99** | 37.3  (35.7, 38.9) | 2 |  | 10 | 22.1  (18.8, 26.0) | 24 | 42.2  (40.4, 44.1) | 6 | 18.2  (15.8, 21.1) |
| RGLF cereals | 6 | 31.4  (29.3, 33.6) | 3 | - | 1 | - | 2 | - | **14** | 36.0  (33.8, 38.3) | 2 | - |
| Discretionary cereals | 1 | 39.2  (34.8, 44.1) | <1 | - | <1 | - | 0 | - | **3** | 40.2  (35.6, 45.3) | <1 | - |
| WGHF breads | 21 | 49.9  (47.7, 52.3) | 6 | 31.5  (28.0, 35.4) | 13 | 44.1  (34.6, 56.4) | **99** | 55.5  (53.5, 57.6) | <1 | - | 6 | 18.8  (16.3, 21.6) |
| RGLF breads | 22 | 52.8  (50.6, 55.2) | 5 | 29.5  (23.2, 37.6) | 36 | 51.3  (45.2, 58.2) | 0 | - | <1 | - | **100** | 55.2  (52.8, 57.8) |
| WGHF grains | 16 | 100.5  (89.3, 113.1) | 14 | 12.6  (9.2, 17.3) | 15 | 25.3  (20.6, 31.0) | 8 | 146.6  (118.5, 181.4) | **27** | 192.0  (180.3, 204.4) | 6 | 109.1  (86.6, 137.4) |
| RGLF grains | 4 | 16.9  (11.0, 26.0) | 1 | - | **26** | 15.7  (10.3, 23.9) | 0 | - | 2 | - | <1 | - |
| Fresh/canned fruit | 24 | 67.5  (60.2, 75.6) | **41** | 37.7  (29.6, 48.0) | 10 | 73.3  (47.8, 112.6) | 14 | 94.0  (74.5, 118.6) | 30 | 87.6  (75.7, 101.4) | 11 | 76.3  (50.6, 115.0) |
| Dried fruit | 20 | 10.0  (9.5, 10.6) | **86** | 9.8  (9.1, 10.5) | 3 | - | 5 | 6.9  (4.6, 10.4) | 6 | 13.6  (10.5, 17.6) | 13 | 10.8  (9.2, 12.7) |
| Brassica vegetables | 4 | 13.1  (9.5, 18.2) | <1 | - | **28** | 16.0  (11.4, 22.4) | <1 | - | <1 | - | <1 | - |
| Orange vegetables | 2 | 11.5  (8.0, 16.4) | <1 | - | **22** | 11.5  (8.0, 16.4) | 0 | - | 0 | - | 0 | - |
| Starchy vegetables | 2 | 14.2  (7.7, 26.3) | <1 | - | **21** | 13.5  (7.2, 25.2) | <1 | - | <1 | - | 0 | - |
| Legumes | 3 | 53.3  (34.1, 83.2) | 3 | - | **8** | 10.0  (5.0, 20.0) | 3 | - | <1 | - | 3 | - |
| All other vegetables | 8 | 42.1  (35.2, 50.4) | 1 | - | **46** | 31.4  (25.0, 39.4) | 11 | 45.2  (35.5, 57.5) | <1 | - | 5 | 89.7  (61.4, 131.1) |
| Reduced fat milks | 37 | 89.5  (84.3, 95.1) | **59** | 109.3  (99.4, 120.2) | 13 | 37.0  (28.7, 47.8) | 35 | 55.3  (48.1, 63.7) | 38 | 130.6  (119.2, 143.1) | 27 | 46.5  (39.1, 55.3) |
| Medium fat milks | 29 | 93.4  (86.0, 101.4) | 25 | 99  (84.1, 116.7) | 29 | 43.2  (31.5, 59.3) | 22 | 60.0  (48.7, 73.9) | **37** | 140.6  (128.4, 153.9) | 24 | 60.4  (52.0, 70.2) |
| Yoghurts & custard | 10 | 76.7  (69.3, 84.8) | **25** | 65.0  57.5, 73.5) | 1 | - | 4 | - | 11 | 95.2  (80.5, 112.5) | 3 | - |
| Cheeses | 5 | 21.9  (19.8, 24.3) | 0 | - | **18** | 18.5  (15.5, 22.0) | 10 | 23.7  (20.6, 27.1) | 1 | - | 7 | 26.7  (21.3, 33.4) |
| Lean red meat | 1 | 25.5  (13.6, 48.0) | 0 | - | **4** | 21.0  (11.1, 39.5) | <1 | - | <1 | - | 0 | - |
| Lean poultry | 1 | 38.1  (25.2, 57.7) | 0 | - | **7** | 38.1  (25.2, 57.7) | 0 | - | 0 | - | 0 | - |
| Fish | 1 | 35.4  (21.5, 58.1) | 0 | - | **9** | 33.4  (19.8, 56.4) | <1 | - | 0 | - | <1 | - |
| Processed meats | 4 | 28.6  (25.8, 31.8) | 0 | - | **26** | 29.1  (25.8, 32.8) | 4 | - | <1 | - | 4 | - |
| Eggs | 9 | 58.7  (54.1, 63.7) | 1 | - | **43** | 56.0  (47.9, 65.5) | 14 | 58.6  (53.2, 64.6) | 2 | - | 6 | 63.3  (53.7, 74.7) |
| Nuts & seeds | 15 | 7.2  (6.6, 7.7) | **49** | 5.4  (4.9, 6.0) | 3 | - | 13 | 9.3  (8.0, 10.8) | 7 | 11.4  (9.2, 14.2) | 7 | 10.5  (8.4, 13.2) |
| Unsaturated oils | 15 | 2.7  (2.5, 2.8) | 27 | 2.2  (2.0, 2.3) | **56** | 3.9  (3.4, 4.4) | 14 | 1.9  (1.7, 2.1) | 2 | - | 8 | 2.0  (1.8, 2.3) |
| Unsaturated spreads | 18 | 5.5  (5.1, 6.0) | 5 | 6.0  (4.4, 8.1) | 36 | 2.1  (1.7, 2.6) | **39** | 6.6  (6.0, 7.3) | <1 | - | 36 | 7.9  (7.2, 8.7) |
| Discretionary spreads | 27 | 10.9  (10.2, 11.7) | 12 | 8.9  (7.4, 10.8) | 11 | 6.9  (5.7, 8.5) | 60 | 10.7  (9.7, 11.8) | 7 | 12.7  (11.2, 14.5) | **62** | 11.8  (10.7, 12.9) |
| Condiments | 2 | 12.0  (9.4, 15.4) | 0 | - | 12 | 11.0  (8.4, 14.5) | 2 | - | <1 | - | 10 | 11.5  (2.7, 49.0) |
| Fruit juice (100%) | 6 | 156.8  (126.6, 194.3) | 8 | 91.6  (47.9, 175.1) | **11** | 108.8  (58.8, 201.3) | 6 | 204.8  (155.3, 270.2) | 5 | 222.6  (181.6, 272.8) | 5 | 216.9  (170.5, 275.9) |
| Water | 21 | 672.6  (616.1, 734.4) | 23 | 642.1  (504.0, 818.2) | **24** | 850.2  (670.4,1078.3) | 16 | 745.7  (584.9, 950.7) | 23 | 657.0  (556.8, 775.3) | 17 | 567.2  (430.8, 746.7) |
| Tea/coffee | 56 | 218.2  (209.4, 227.3) | 55 | 223.6  (202.1, 247.5) | 41 | 220.6  (192.9, 252.3) | 66 | 211.3  (199.7, 223.6) | 51 | 221.2  (208.2, 235.1) | **68** | 215.3  (193.4, 239.6) |
| Sugar | 23 | 7.3  (6.9, 7.7) | 13 | 6.1  (5.2, 7.2) | 15 | 6.3  (4.9, 8.0) | 22 | 5.8  (5.2, 6.4) | 27 | 8.9  (8.1, 9.8) | **30** | 6.9  (6.3, 7.5) |
| SSBs | 5 | 79.9  (54.8, 116.5) | 3 | - | **7** | 122.0  (74.7, 199.5) | 4 | - | 5 | 55.0  (28.9, 104.7) | 5 | 73.4  (36.2, 148.9) |
| Sweet cereal products | 2 | 47.2  (40.0, 55.7) | 1 | - | 4 | - | 1 | - | **5** | 56.2  (48.4, 65.2) | <1 | - |
| Savoury cereal products | 2 | 56.1  (43.7, 72.0) | 0 | - | **7** | 45.4  (30.6, 67.5) | 0 | - | 2 | - | 0 | - |

^1^Values shown are weighted percentage (%) of women who reported consuming one or more food/beverage items from each food group at the breakfast eating occasion and weighted geometric mean (95% confidence interval) intake of food groups where percentage of consumption for a latent breakfast profile was: ≥ 5%, or the highest percentage for less frequently consumed foods groups (e.g., Discretionary cereals; Lean red meat). Values in bold indicate the highest proportion of consumption across breakfast profiles for each food group. Abbreviations: RGLF, refined grain or lower fibre; SSBs: sugar-sweetened beverages; WGHF, wholegrain or high fibre
